# *CUsKit*: a test to revolutionise the evaluation of the disinfection process

**DOI:** 10.1007/s00253-025-13456-8

**Published:** 2025-03-22

**Authors:** Débora Castro, Isabel Ferreri, Mariana Henriques, Isabel Carvalho

**Affiliations:** 1 CEB, Centre of Biological Engineering, University of Minho, Campus of Gualtar, Braga, 4710-057 Portugal; 2 Success Gadget-Nanotecnologia e Novos Materiais, Lda, Rua Filipa Borges, 1245, 4750-823Barcelos Braga, Portugal; 3 LABBELS–Associate Laboratory, Braga/Guimarães, Portugal

**Keywords:** Detection kit, Hydrogen peroxide, Antimicrobial activity

## Abstract

**Abstract:**

The overuse of surface disinfectants has been shown to lead to increased microbial resistance, often due to their incorrect application. The aim of this work was to develop and investigate the use of an indicative detection kit for hydrogen peroxide-based disinfectants on porous and non-porous surfaces. The detection kit allows for the detection of the disinfectant up to 7 days after its application, resulting in a colour change from transparent to yellow-orange on both on porous and non-porous surfaces. Concurrent antimicrobial tests were performed to establish the correlation between the colour change and the retention of the disinfectant on the surfaces, thereby confirming the maintenance of its antimicrobial efficacy.

**Key points:**

- *Increased microbial resistance often results from overuse of disinfectants.*
- *CUsKit is a hydrogen peroxide-based disinfectant detection kit.*
- *CUsKit is an excellent option to prove the presence of disinfectants.*

## Introduction

Chemical disinfectants have been widely used during the pandemic, caused by severe acute respiratory syndrome coronavirus-2 (SARS-CoV-2) the virus that causes coronavirus disease (COVID-19) (Chen et al. 2021; Musee et al. 2023; Jia et al. 2023). A concerted effort has been made to control the spread of the virus with local authorities, governments, and public health institutions around the world to implement effective preventive measures, particularly on commonly touched surfaces in public places (Musee et al. 2023; Chen et al. 2021; Mc Carlie et al. 2020). Since then, there has been an increase in disinfection campaigns in public facilities, shared community spaces, and homes, through hand washing and regular decontamination of surfaces (Musee et al. 2023; Al-Sayah 2020; Kim et al. 2018; Mc Carlie et al. 2020). This type of behaviour has been recommended by the public health agencies, such as the World Health Organization (WHO) (Al-Sayah 2020).

Disinfectants are extensively employed for the purpose of sterilising surfaces and spaces, an area, or a device. The sterilising effect of a disinfectant is characterised by its ability to either eliminate or causes irreversible inactivation of infectious viruses, germs, and bacteria on surfaces or objects (Al-Sayah 2020; Musee et al. 2023; Chen et al. 2021). In fact, disinfectants play a vital role in the ecological health and safety of life and have potential applications in medical fields, water treatment and distribution, food processing, agricultural industries, healthcare facilities, and other areas (Tong et al. 2021; Kim et al. 2018).

The fundamental characteristic of an ideal disinfectant is that it should require a brief contact time while exhibiting significant antimicrobial activity. The capacity of a disinfectant is contingent upon its mode of chemical action, the molecular structure of the surface of the pathogen, and its intracellular susceptibility (Pradhan et al. 2020; Al-Sayah 2020; Carvalho et al. 2013).

Given the need to reduce the spread of microorganisms on surfaces, the lack of scientific management and strategic planning has led to the overuse and misuse of disinfectants in many public areas, resulting in a reduction in disinfection efficacy (Tong et al. 2021; Chen et al. 2021; Pradhan et al. 2020; Adhikari 2014; Mc Carlie et al. 2020; Jia et al. 2023). This phenomenon is of particular concern as antimicrobial resistance is emerging at an accelerated rate, posing a significant threat to public health (Mc Carlie et al. 2020; Adhikari 2014).

The extensive and indiscriminate use of chemical disinfectants has allowed bacteria to evolve and withstand exposure to these agents. Another major concern is the potential negative ecological impact of such practices (Chen et al. 2021). In addition, the widespread use of toxic and corrosive disinfectants in urban outdoor environments poses a significant threat to urban wildlife and aquatic environments (Chen et al. 2021). In this context, antimicrobial resistance emerged from regularly disinfected surfaces and surrounding environments that are regularly disinfected, and this in the vicinity and became a real risk, due to the significant reduction in the killing efficiency of disinfectants (Chen et al. 2021; Tong et al. 2021).

The widespread use of surface disinfectants, often incorrectly, without ensuring the necessary contact time with the surface to be treated or due to insufficient product availability leads to an escalation of microbial resistance. In view of these concerns, and with the aim of regulating the use of disinfectants, this work presents a novel detection kit for hydrogen peroxide-based disinfectants, to detect the presence of disinfectant on different surfaces (porous or non-porous) after its application.

This detection kit for hydrogen peroxide-based disinfectants is denominated by *CUsKit*, it is a quick and intuitive kit that can be used by anyone, whether at a personal or professional level, without the need for training. *CUsKit* is ground-breaking because, to our knowledge, it is the first kit to detect hydrogen peroxide on any surface, porous or non-porous. All studies on the detection of hydrogen peroxide have been performed in liquid media, such as household disinfectants (Zhang et al. 2022), milk samples (Zhang et al. 2021), various water samples (Guo et all., 2017; Qin et al. 2018; Xiong et al 2017), human serum (Gong et al 2017), and neutral solutions (Muralikrishna et al 2016). The indicator solution contained in the kit, which consists of potassium iodide, turns an orange colour when it comes into contact with hydrogen peroxide-based disinfectants. This orange colour results from the reaction of potassium iodide with hydrogen peroxide, according to the following chemical Eq. 1 (Ciesielski et al., 2006).

1 $$KI+H_2O_2\rightarrow KIO+H_2O$$

**Fig. 1:**
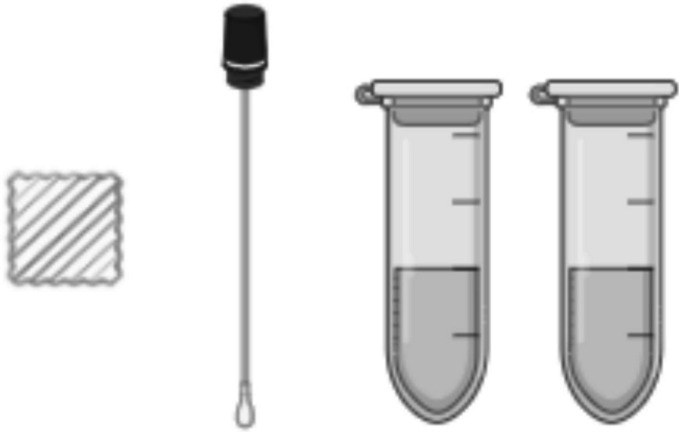
Schematic representation of the *CUsKit* components

The *CUsKit* consists of a textile sample (representative of porous surfaces), to which the disinfectant is applied at the same time as the surface to be treated, a swab to be used on non-porous surfaces, and two Eppendorf, with a transparent indicator solution to dip the swab and textile sample (Fig. 1).

## Material and methods

### Evaluation of the applicability of *CUsKit* with different solutions

The hydrogen peroxide-based disinfectant detection kit test was performed with 5 different solutions: solution A, hydrogen peroxide textile disinfectant; solution B, commercial solution of hydrogen peroxide at 49.5% (w/w) diluted to a concentration of 3% (w/w); solution C, antiseptic solution of hydrogen peroxide at a concentration of 3% (w/w); solution D, multi-surface disinfectant based on hydrogen peroxide at a concentration of 1.49% and the solution E, multi-surface disinfectant without hydrogen peroxide. For this purpose, swabs were used, each swab placed in the Eppendorf tube containing the kit’s indicator solution and after removal, brought into in contact with solutions A to E.

To further refine the study, it was decided to use solution D, called *Care Us*, supplied by the company Success Gadget, Nanotecnologia e Novos Materiais, Lda. This particular solution contains hydrogen peroxide as the main active ingredient and has been shown to provide antimicrobial activity for 7 days after application (Castro et al. 2022).

### Evaluation of *CUsKit* on porous surfaces

Textile sample (2.0 × 2.0) cm^2^ used in the *CUsKit* were provided by a local textile industry (Malhas Sonix, S.A., Barcelos, Portugal) and consists of a single jersey of 137 g/m^2^, 30/1, 100% cotton, BCI (Better Cotton Initiative), set 28, with optical brightener.

**Fig. 2:**
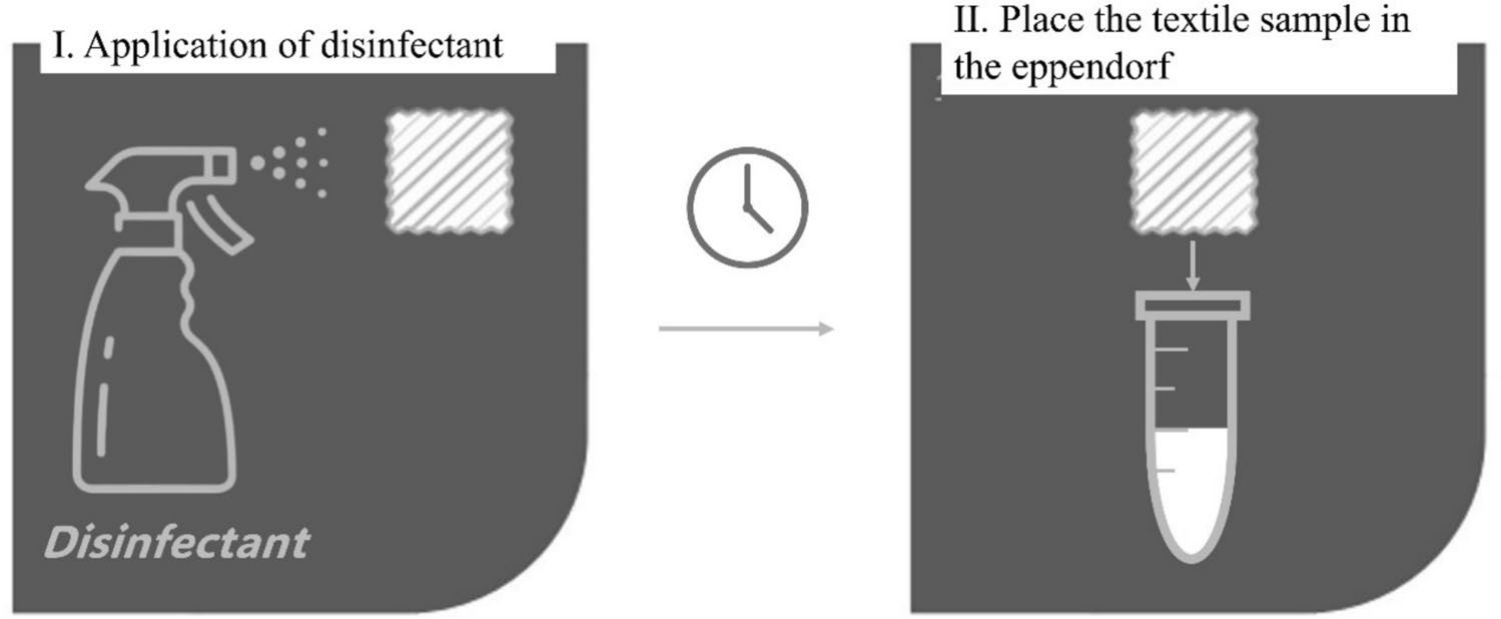
Schematic representation of the use of *CUsKit*, on porous surfaces

For the 7-day tests with solution D, nine textile samples were used: one sample as a control (no disinfectant applied) and eight samples for each day (day 0 to day 7). The eight textile samples were treated with disinfectant on day 0 and then one sample was analysed in each day by placing it an Eppendorf with the indicator solution (Fig. 2).

The quantification of hydrogen peroxide was conducted in accordance with the standard IS:7045–1973 (Indian Standards, 1973). The textile samples under study were quantified by means of a titration method.

### Evaluation of *CUsKit* on non-porous surfaces

**Fig. 3:**
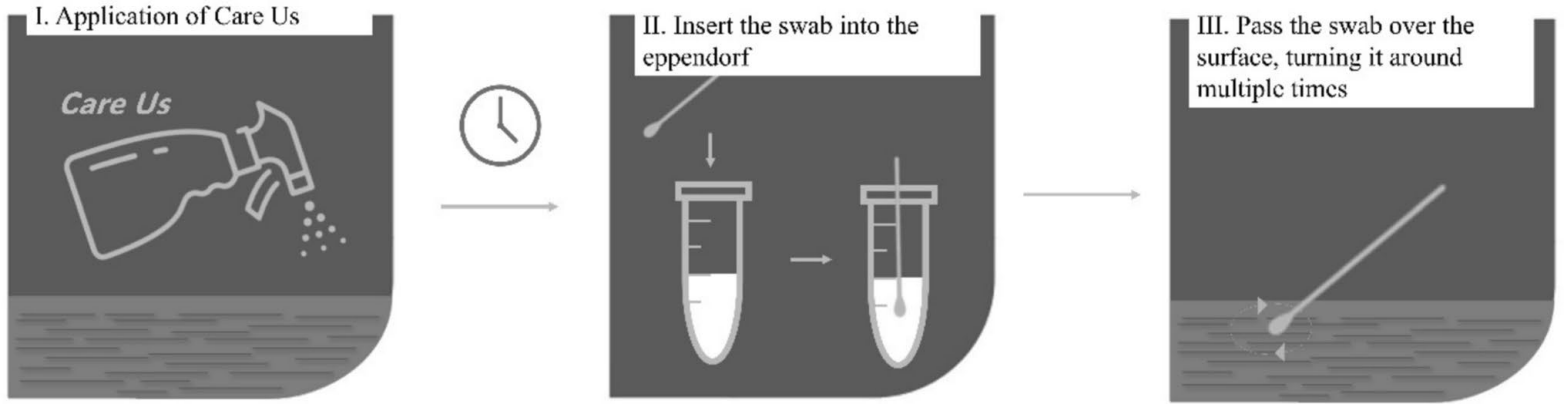
Schematic representation of the use of *CUsKit*, on non-porous surfaces

The presence of disinfectant on non-porous surfaces was investigated using a glass surface. Nine squares (10.0 × 10.0) cm^2^ were marked on the glass surface, one for the control (no disinfectant applied) and eight for the day after the disinfectant was applied (day 0 to day 7) The eight squares corresponding to days 0 to 7 were treated with the disinfectant on day 0. On each day, a test swab was dipped into the indicator solution and after removing it, it was rotated on the surface corresponding to the day under study, both parallel and perpendicular to its own orientation, for a period of 20 s. The procedure is illustrated in Fig. 3.

### Accelerated stability test of indicator solution

The accelerated stability test was conducted in accordance with a methodology delineated by CIPAC (Collaborative International Pesticides Analytical Council), a widely accepted protocol within the cosmetics industry (CIPAC 2000). The formulation is subjected to elevated temperatures for a limited duration. This extreme time–temperature binomial has been shown to simulate the normal aging of a formulation (CIPAC 2000). The 14-day period at 54 °C in this test is equivalent to 2 years at ambient temperature in terms of shelf life (CIPAC 2000).

The objective of this test is to provide actionable guidelines on the performance of the product by analysing its chemical and physical characteristics. It is imperative to note that this test is mandatory to predict the stability of the formulation and its shelf life.

In this study, accelerated stability testing was employed to ensure that the potassium iodide indicator solution remained stable and effective throughout the kit’s 2-year shelf life. The indicator solution is placed in an oven (*Memmert*) at 54 ± 2 °C for 14 days, which can be equivalent to a shelf life of 2 years (CIPAC 2000). For the present study, approximately 40 mL of the indicator solution was exposed to a temperature of 54 ± 2 °C for a period of 14 days.

Following a 14-day period of accelerated stability testing, the detection kit was used on both surfaces in a manner consistent with the description provided in the preceding section. This was done to ascertain whether the solution retained its capacity to detect the presence of hydrogen peroxide-based disinfectant.

### Biological analyses

#### Evaluation of antimicrobial activity on non-porous surfaces

The primary function of the detection kit is to minimise the use of disinfectants. Consequently, it is imperative to ensure that the colour change of the test is indicative of antimicrobial activity of the disinfectant. The procedure for these tests has been adapted from the ISO 18593:2004 standard (ISO 18593, 2004).

Nine zones were demarcated, referring to control and the time after application of the disinfectant (day 0 to day 7). The zones were demarcated with adhesive tape, with each zone having a surface area of 100 cm^2^ (ISO 18593, 2004). In the control zone, no disinfectant was applied, while in the other zones, the disinfectant was applied in the same way and at the same time (day 0). From day 0 to day 7, samples were taken using a swab moistened in a tube containing approximately 2 mL of phosphate-buffered saline 1 × (PBS 1 ×). The swab was then moved in a parallel and perpendicular direction to the surface, with constant rotation to ensure thorough sampling (ISO 18593, 2004). The swab was then transferred to a Petri dish containing Tryptone Soy Agar (TSA) (purchased from Frilabo), inverted, and left at room temperature for 168 h to analyse the appearance of bacteria, fungi, and yeasts (ISO 18593, 2004). This experiment was replicated in at least three independent experiments. It should be noted that this test was carried out against native microorganisms present on the test surface; no test organisms were seeded on the surface.

## Results

### Evaluation of applicability of the *CUsKit*

**Fig. 4:**
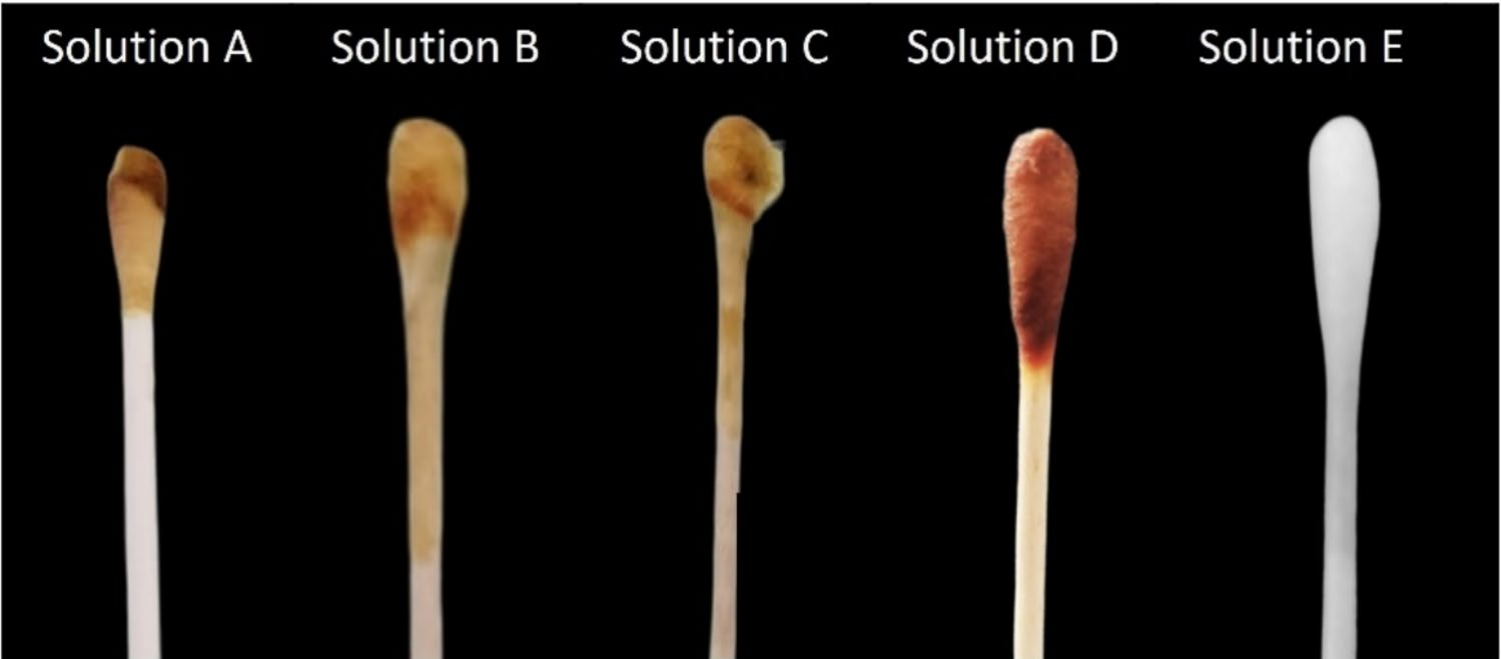
Use of the detection kit in different solutions containing hydrogen peroxide (solutions A, B, C, and D), and a negative control without hydrogen peroxide (solution E)

Five solutions were used for the evaluation of the kit. Figure 4 shows a colour formation in all the solutions containing hydrogen peroxide (solutions A, B, C, and D), with the exception of solution E, which was employed as a negative control, given its lack of hydrogen peroxide.

### Evaluation of *CUsKit* on porous surface

Solution D, *Care Us*, was chosen to perform this test and the following ones.

**Fig. 5:**
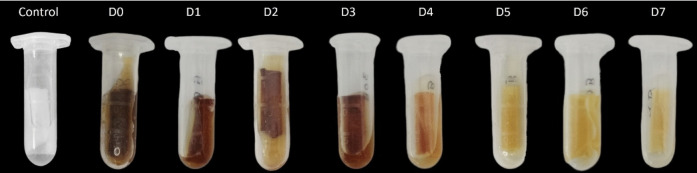
Results of the *CUsKit*, on porous surfaces, after the application of disinfectant, on different days (D)

Textile samples are representative samples of porous surfaces used in the context of disinfectant detection testing. The test was carried out over a period of 7 days with the aim of detecting the disinfectant at each time after its application to the textile (from day 0 to day 7) through the colour change of the indicator solution, as can be seen in Fig. 5.

**Fig. 6:**
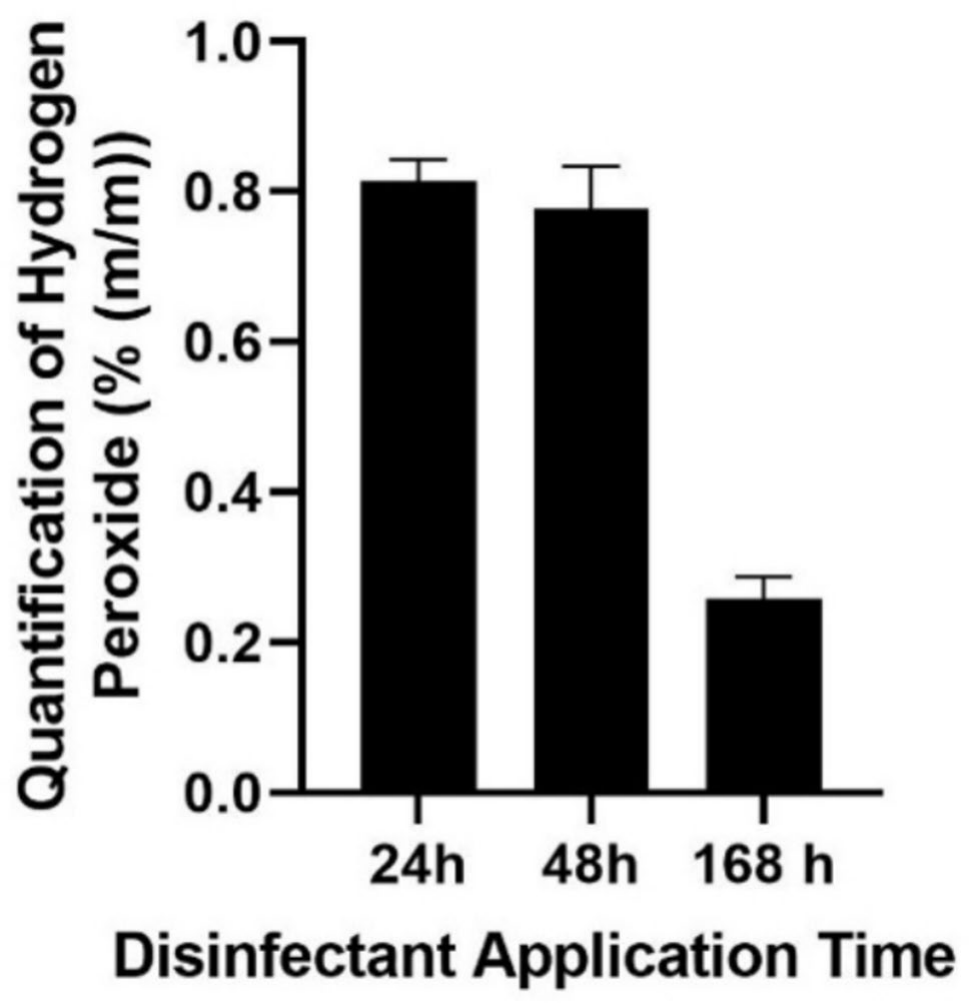
Quantification of hydrogen peroxide, by iodometry, as a function of disinfectant application time, over textile samples (Indian Standard 1973)

As shown in Fig. 5, the indicator solution changed colour indicating the presence of disinfectant in the textile samples. However, a loss of colour was observed over time as the interval between application of the disinfectant and the test increased, i.e. a greater loss of colour was observed 7 days after application. In order to correlate the colour loss of *CUsKit* on porous surfaces with the loss of disinfectant over time, iodometric quantification of hydrogen peroxide was performed on the textile samples, as shown in Fig. 6.

A comparison of the time points between 24 and 168 h, as illustrated in Fig. 6**,** shows a substantial decline in the active substance, corresponding to a loss of 68.3% of the initial amount of hydrogen peroxide.

### Evaluation of *CUsKit* on non-porous surface

**Fig. 7:**
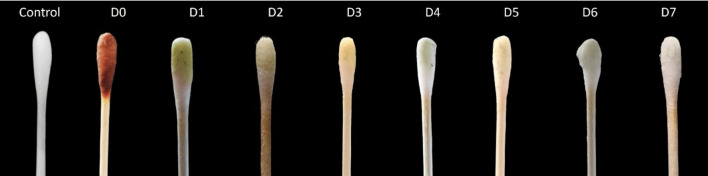
Results of performing the *CUsKit*, on non-porous surfaces, on different days (D) after the application of the disinfectant

The *CUsKit* was evaluated on a glass surface, which is representative of a non-porous surface. The detection test procedure described above was carried out and the results are shown in Fig. 7.

The test procedure involved the adsorption of an indicator solution on a swab, which subsequently underwent a colour change, indicative of the presence of a disinfectant on the glass surface. The colour change was most pronounced up to day 5, with yellow areas appearing on the swab. A colour change was also observed on the last 2 days, although it was less pronounced when photographed.

### Accelerated stability of indicator solution

**Fig. 8:**
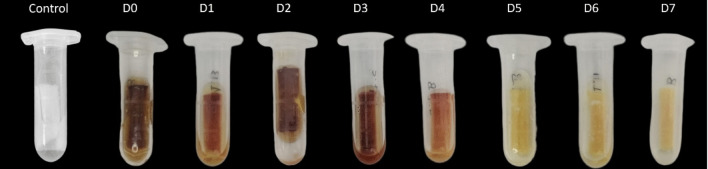
Results with the *CUsKit*, after accelerated stability tests of the indicator solution, to determine whether it maintains its detection properties

In the accelerated stability test, the indicator solution was placed in an oven and exposed to a temperature of 54 ± 2 °C, for a period of 14 days. At the end of this period, the disinfectant detection test was performed and the results obtained are displayed in Fig. 8.

As shown in Fig. 8, the indicator solution retains its ability to detect disinfectant on a porous surface after a 7-day of application, following the “accelerated ageing” process in the oven, as evidenced by the colour change of the indicator is observed over the course of 7 days.

### Biological assays

**Fig. 9:**
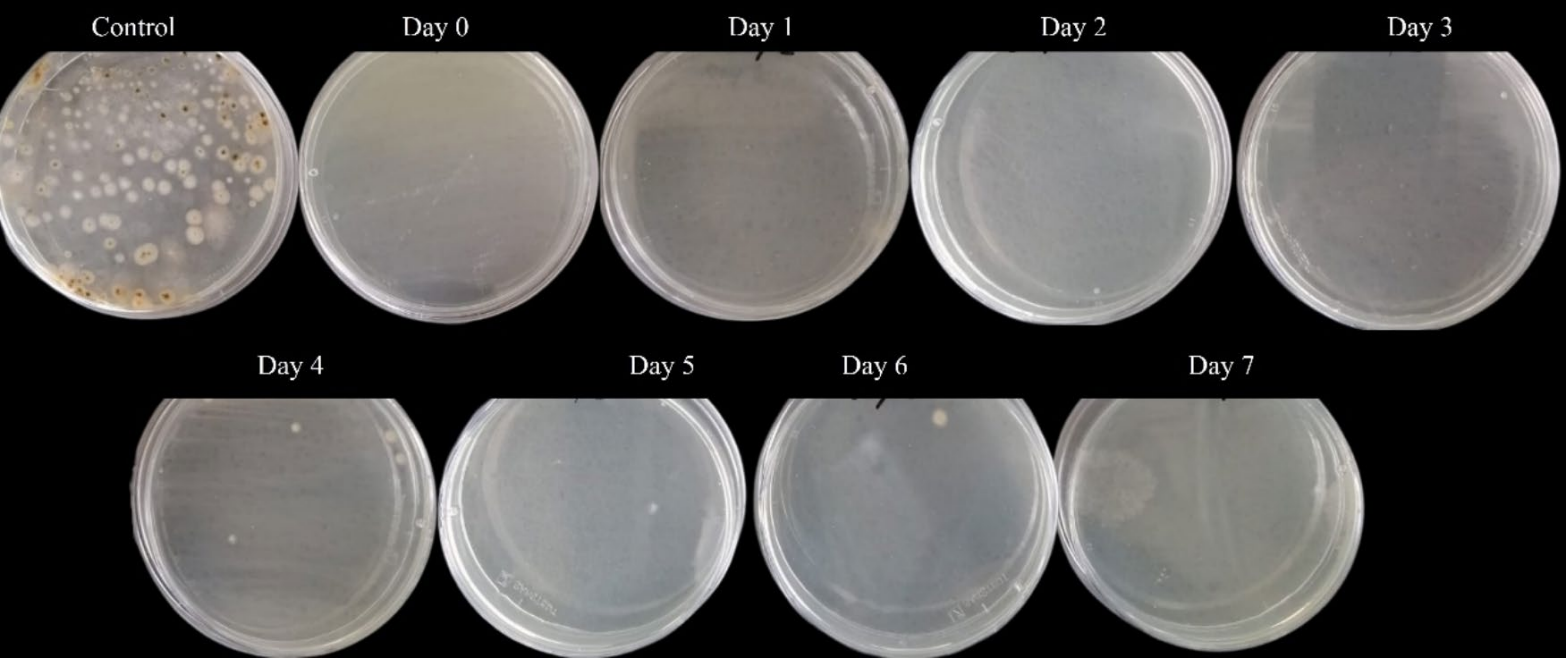
Results of test on everyday surfaces: control sample, and samples with application of disinfectant, in different days

Concurrently with the *CUsKit* test on non-porous surfaces, antimicrobial tests were carried out on the same surfaces. The ISO 18593:^2004^ standard was therefore used, and the results are shown in Fig. 9.

Figure 9 shows a substantial inhibition of microbial growth in all samples treated with disinfectant.

## Discussion

### Evaluation of the applicability of *CUsKit* with the different solutions

The aim of this study was to investigate the use of an indicative detection kit for hydrogen peroxide-based disinfectants to reduce the use of such products. A specific test, designated *CUsKit* was developed and validated with a suitable indicator solution for the active ingredient hydrogen peroxide.

The *CUsKit* was effective against a range of disinfectants and hydrogen peroxide solutions, demonstrating its high performance.

As the kit gave positive results for all the solutions, solution D was selected for further testing as it had already been fully tested and guaranteed antimicrobial activity for 7 days, on both porous and non-porous surfaces (Castro et al. 2022).

### Evaluation of *CUsKit* on porous surface

Figure 5 shows a colour change in the indicator solution, indicating the presence of disinfectant in the textile samples. This colour change is due to a chemical reaction between hydrogen peroxide (H_2_O_2_) and potassium iodide (Ciesielski et al., 2006). The decomposition of the hydrogen peroxide present in the textile samples is catalysed by the potassium iodide from the indicator solution, as demonstrated in Eq. 1.The colour change of the indicator solution becomes less pronounced as the time between the application of the disinfectant and the test increases but is still remains visible in the final days of the test. This slight colour change in the indicator solution is a normal occurrence as some disinfectant products are naturally lost from surfaces over time. Figure 6 illustrates the relationship between the loss of hydrogen peroxide and the time after the application of the disinfectant.

The variation in time points between 24 and 168 h, shown in Fig. 6, is attributed to the decomposition of the active ingredient (hydrogen peroxide) into water and oxygen over time. This reaction is accelerated by exposure to sunlight and high temperatures, which are common in everyday life.

### Evaluation of *CUsKit* on non-porous surface

The change in colour of the indicator solution previously adsorbed on the swab, seen in Fig. 7, indicates the presence of disinfectant on the glass surface. This change is more pronounced up to the fifth day due to the loss of hydrogen peroxide over the time, as previously observed on porous surfaces. However, this change in colour is more pronounced in the test carried out on porous surfaces than in the test on non-porous surfaces. This is due to the methodology, since the textile treated with the disinfectant comes into direct contact with the indicator solution, whereas on non-porous surfaces the contact is indirect, carried out by means of a swab.

The colour change on porous surfaces is fully detectable up to 7 days after application, while on non-porous surfaces it is more noticeable up to 5 days after disinfectant application.

### Accelerated stability of indicator solution

After the “accelerated ageing” process in the oven, the colour change of the indicator is still observed even after a period of 7 days, as seen in Fig. 8. These results are very similar to those shown in Fig. 5, where *CUsKit* was subjected to normal temperature conditions (room temperature). Consequently, the shelf life of the indicator solution is 2 years according to the CIPAC method (CIPAC 2000).

### Biological assays

The control sample exhibited a substantial presence of bacterial and fungal colonies, as seen in Fig. 9, representative of a surface that had not been subject to the application of disinfectant. In contrast to the significant decrease in the bacterial and fungal load of the samples, treated with the disinfectant.

Previous studies carried out with disinfectants on non-porous surfaces, under aseptic conditions and in accordance with the ISO 22196:2011 standard, have shown remarkable antimicrobial efficacy (Castro et al. 2022). In this present study, the surfaces actually analysed were glass surfaces, classified as non-porous surfaces, which suggests that these results are comparable to those obtained in previous tests.

The results shown in Fig. 9 demonstrate that the disinfectant is able to significantly restricting microbial growth on all samples treated with the disinfectant for a maximum period of 7 days when applied to everyday surfaces. This suggests that the need for frequent and recurrent disinfection procedures can be reduced during this period. The reduction in colour change on non-porous surfaces after 5 days is not accompanied by a loss of antimicrobial activity, thus maintaining excellent results.

The antimicrobial tests carried out demonstrate that the colour change of the indicator solution in the detection kit is associated with the presence of the disinfectant and therefore with its antimicrobial activity.

The importance of the detection kit lies in its capacity to provide a straightforward method of correlating the optical presence of the hydrogen peroxide-based disinfectant with the absence of microbial growth, thereby ensuring the effectiveness of the disinfection process.

Consequently, it can therefore be concluded that *CUsKit* is a highly effective option to demonstrate the efficacy of disinfectants in a straightforward and cost-effective manner.

## Data Availability

The author confirms that all data generated or analysed during this study are included in this published manuscript. Furthermore, primary and secondary sources and data supporting the findings of this study were all publicly available at the time of submission.

## Declarations

### Disclaimer

This manuscript does not contain any studies with human participants performed by any of the authors.
